# Influence of Different Levels of a Rubber Seed Kernel Pellet Supplement on Feed Efficiency, Blood Chemistry, and Growth Performance in Beef Cattle

**DOI:** 10.1155/vmi/2226359

**Published:** 2026-07-30

**Authors:** Pongsatorn Gunun, Walailuck Kaewwongsa, Natthapong Morthong, Ruttanachira Ruttanaprasert, Chatchai Kaewpila, Waroon Khota, Nirawan Gunun

**Affiliations:** ^1^ Department of Animal Science, Faculty of Natural Resources, Rajamangala University of Technology Isan, Sakon Nakhon Campus, Sakon Nakhon 47160, Thailand, rmuti.ac.th; ^2^ Department of Animal Science, Faculty of Technology and Engineering, Udon Thani Rajabhat University, Udon Thani 41000, Thailand, udru.ac.th; ^3^ Udon Thani Provincial Livestock Office, Udon Thani 41000, Thailand; ^4^ Department of Plant Science, Textile and Design, Faculty of Agriculture and Technology, Rajamangala University of Technology Isan, Surin Campus, Surin 32000, Thailand, rmuti.ac.th

**Keywords:** blood metabolites, byproduct pellet, growth performance, tropical beef cattle

## Abstract

The rubber seed kernel is a byproduct of rubber plantations and has potential as a source of protein and lipids. The aim of the current study was to evaluate the effect of rubber seed kernel pellet (RUSKEP) supplementation on feed intake, nutrient digestibility, blood chemistry, and growth performance. Twelve crossbred (Brahman × Thai native) bulls with a body weight (BW) of 285 ± 17 kg were randomly assigned to three treatments for 60 days in a completely randomized design (CRD). RUSKEP was supplemented at 0, 200, and 400 g/animal/day. Animals were fed a total mixed ration (TMR) at 2.3% BW. The dry matter (DM) intake was similar among treatments (*p* > 0.05). The addition of RUSKEP did not influence nutrient intake (*p* > 0.05), whereas ether extract (EE) intake increased with the increasing levels of RUSKEP (*p* < 0.001). The digestibility of crude protein (CP) and EE increased with increasing levels of RUSKEP (*p* ≤ 0.03). Supplementation of RUSKEP had no effect on blood metabolites (*p* > 0.05), except for cholesterol, which increased with adding RUSKEP (*p* ≤ 0.005). Average daily gain (ADG) increased with the RUSKEP supplementation (*p* = 0.03). ADG was highest at 200 g/animal/day, suggesting a potential optimal level for cattle. The feed conversion ratio (FCR) decreased with the RUSKEP supplementation (*p* = 0.04). Supplementation with 200 g/animal/day of RUSKEP improves ADG and feed efficiency in tropical beef cattle.

## 1. Introduction

Energy and protein supply are the major limiting factors that affect the productivity of livestock production [1]. In tropical countries, most cattle are fed low‐quality roughage and commonly used byproducts in their diet [2]. This, in turn, leads to a decrease in feed utilization, rumen fermentation, and ultimately, a reduction in growth performance [3, 4]. The supplementation of local feed ingredients as a high protein and lipid source has the potential to enhance feed efficiency and animal performance [5, 6]. In economic terms, feed is the most important aspect of beef production, as feed costs can constitute up to 70% of the total production costs [7]. Therefore, it is important to enhance the utilization of nutrients in order to increase the positive impact in terms of both cattle and the economy’s profitability [8].

Many tropical countries, especially Thailand, cultivate the rubber tree (*Hevea brasiliensis*), which is the main source of natural rubber production [9]. The rubber seed is a byproduct of the rubber tree, which has an average of 150 kg of seeds per hectare per year [10], and rubber seed availability is estimated at 0.34 million metric tons per year [11]. A rubber seed consists of a kernel (60%) and a shell (40%) [12]. The rubber seed kernel contains 21.2% crude protein (CP), 34.3% ether extract (EE), and 8030 kcal/kg dry matter (DM) gross energy (GE) [11]. The rubber seed kernel is recognized as a source of polyunsaturated fatty acids (PUFAs), containing 39.3%–40.3% linoleic acid (LA; C18:2 cis‐9,12 + trans‐9,12) and 21.2%–23.4% α‐linolenic acid (ALA; C18:3 cis‐9,12,15) [13]. Consequently, these byproducts have the potential to be used as protein and lipid sources for ruminant diets. The inclusion of fermented rubber seed kernel with yeast at 10%–25% in concentrated diets did not influence feed efficiency, rumen fermentation characteristics, or milk yield in dairy cattle [12, 14]. An in vitro study also showed that adding heat‐processed rubber seed kernel to TMR diets improves the bacterial population and lowers the biohydrogenation of C18 unsaturated fatty acids (UFAs) in the rumen [13]. In addition, the dietary supplementation of rumen‐protected rubber seed oil improved the digestibility, milk yield, and milk fat with PUFA, especially LA and ALA, in dairy cows [15].

The feed pellets have been optimized to prevent nutrient segregation, reduce feed spoilage, enable more efficient transportation and storage, enhance feed availability, and lead to increased livestock production [16–18]. Our earlier research showed that adding up to 10% rubber seed kernel pellet (RUSKEP) improved the in vitro rumen fermentation and the amount of PUFA, especially ALA, in the rumen [19]. The addition of RUSKEP, up to 8% of DM intake, enhanced rumen fermentation patterns while having no effect on feed availability, rumen PUFA content, or immunity in swamp buffalo [20]. Nevertheless, there is limited data on growth performance and feed utilization responses in beef cattle production. We hypothesize that the addition of RUSKEP at appropriate levels could improve feed availability and growth performance. The objective of this study was to examine the influence of RUSKEP supplementation on feed intake, digestibility, blood parameters, and growth performance in tropical beef cattle.

## 2. Materials and Methods

### 2.1. Ethical Procedure

The authors confirmed that all methods were carried out in accordance with the applicable guidelines and regulations. Animal care obtained approval from the Animals Ethical Committee of Udon Thani Rajabhat University (approval number 0622.7/103). The study was carried out in compliance with the ARRIVE guidelines.

### 2.2. Preparation of RUSKEP

Experimental research and field studies on plants (either cultivated or wild), including the collection of plant material, must comply with relevant institutional, national, and international guidelines and legislation. The *Hevea brasiliensis* (rubber) tree belongs to the family Euphorbiaceae. The seeds were collected by hand from the ground and stored indoors. A dehulling machine (Incanewlife, Khon Kaen, Thailand) removed the shells from the seeds. The kernels were dried in sunlight for 3 days, followed by grinding to pass through a 1‐mm sieve. The RUSKEP was prepared by using rubber seed kernel (82.00%), cassava starch (7.00%), *Tinospora cordifolia* stem powder (5.00%), molasses (3.00%), mineral mixture (1.00%), vitamin mixture (1.00%), or salt (1.00%) (Table 1) and then making the mixture into pellets using a pellet machine. Pellets were made from rubber seed kernels using a 5‐mm diameter die, resulting in bulk density values of 0.56 g/cm^3^.

**TABLE 1:** Ingredients and chemical compositions of rubber seed kernel pellet (RUSKEP).

Item	RUSKEP
Ingredient, kg DM	
Rubber seed kernel meal	82.00
Cassava starch	7.00
*Tinospora cordifolia* stem powder	5.00
Molasses	3.00
Mineral mixture	1.00
Vitamin mixture	1.00
Salt	1.00
Chemical composition	
Dry matter, %	89.58
Organic matter, %DM	94.43
Crude protein, %DM	16.50
Ether extract, %DM	26.11
Neutral detergent fiber, %DM	28.70
Acid detergent fiber, %DM	19.91
Fatty acid, % of total fatty acid	
C16:0	10.01
C17:0	0.07
C18:0	9.14
C20:0	0.32
C22:0	0.06
C23:0	0.09
C16:1 cis‐9	0.21
C18:1 cis‐9 (OA)	24.38
C18:2 cis‐9,12 + tran‐9,12 (LA)	40.16
C18:3 cis‐9,12,15 (ALA)	15.19
C20:1 cis‐11 (EA)	0.10
C20:5 cis‐5,8,11,14,17 (EPA)	0.16
C22:1 cis‐13	0.12
SFA	19.69
UFA	80.31
MUFA	24.81
PUFA	55.50

Abbreviations: ALA, α‐linolenic acid; DM, dry matter; EA, eicosanoic acid; EPA, eicosapentaenoic acid; LA, linoleic acid; MUFA, monounsaturated fatty acids; OA, oleic acid; PUFA, polyunsaturated fatty acid; SFA, saturated fatty acids; UFA, unsaturated fatty acids.

### 2.3. Experimental Design and Dietary Treatments

This research was carried out at a Rai Phu‐Tawan cattle farm, Udon Thani, Thailand. Twelve crossbred (Brahman × Thai native) bulls with a BW of 285 ± 17 kg were conducted using a CRD to receive three treatments. The TMR had been created to be isonitrogenous, comprising 10.5% CP on a DM basis, and isocaloric, with a metabolizable energy (ME) of 11.7 ME MJ/kg DM, in accordance with the recommended nutrient requirements [21]. The forage to concentrate ratio of TMR was 20:80 in DM. The TMR consisted of cassava chip (56.11%), brewers’ grains (16.00%), rice straw (9.00%), Napier grass (6.22%), soybean meal (5.29%), corn silage (5.00%), mineral and vitamin mixture (1.38%), salt (0.83%), and sulfur (0.17%) (Table 2). The cattle were fed TMR at 2.3% BW. The supplementation of RUSKEP was 0, 200, and 400 g/animal/day, respectively. The animals were fed twice a day at 08:00 and 17:00 h. The cattle were always housed in individual pens (3 × 6 m), with concrete flooring and a steel fence. The animals received access to clean water.

**TABLE 2:** Ingredients and chemical composition of the diet used in the experiment.

Item	TMR
Ingredient, kg DM	
Cassava chip	56.11
Brewers’ grains	16.00
Rice straw	9.00
Napier grass	6.22
Soybean meal	5.29
Corn silage	5.00
Mineral and vitamin mixture	1.38
Salt	0.83
Sulfur	0.17
Chemical composition	
Dry matter, %	46.06
Organic matter, % DM	87.77
Crude protein, % DM	10.50
Ether extract, % DM	2.01
Neutral detergent fiber, % DM	32.60
Acid detergent fiber, % DM	19.19
ME, MJ/kg DM^1^	11.70

Abbreviations: DM, dry matter; ME, metabolizable energy; TMR, total mixed ration.

^1^Calculate value.

### 2.4. Data Collection and Sampling Procedures

The cattle received a 14‐day adaptation period to their experimental diet prior to the initiation of the 60‐day experimental period. The ADG was assessed by weighing cattle at the initial BW, 30 days, and final BW at 60 days. Daily measurements of refusal weights were taken and recorded prior to the morning feeding to determine DM intake.

The RUSKEP, TMR, and refusal samples were gathered every week, combined, and kept at −20°C until analysis. The samples were dried at 60°C in a hot air oven and ground (1‐mm screen using Cyclotech Mill; Tecator, Hoganas, Sweden). The DM, ash, and EE contents have been investigated in accordance with AOAC [22]. The CP content was measured using an N analyzer (828 Series, LECO, St. Joseph, MI, USA). The neutral detergent fiber (NDF) and acid detergent fiber (ADF) were measured using the fiber analyzer (ANKOM 200, ANKOM Technology, NY, USA) [23]. The fatty acid compositions in RUSKEP were evaluated in accordance with Christie [24] using gas chromatography (GC 8890; Agilent Technologies Ltd., Santa Clara County, CA, USA).

During the last 5 days of the trial (Days 56–60), we collected feces (about 500 g) from each animal through rectal sampling in the morning (07:00 h) and afternoon (16:00 h) [4]. The daily fresh feces of each animal were collected and subsequently kept in the refrigerator at 4°C. Two consecutive samples were mixed together, and the composite was thereafter stored in the refrigerator at −20°C until analysis. The feces samples were dried at 60°C in a hot air oven and ground (1‐mm screen using Cyclotech Mill; Tecator, Hoganas, Sweden). The DM, ash, and EE contents have been determined in accordance with AOAC [22]. The CP content was measured using an N analyzer (828 Series, LECO, St. Joseph, MI, USA). The NDF and ADF were measured using the fiber analyzer (ANKOM 200, ANKOM Technology, NY, USA) [23]. The evaluation of the acid‐insoluble ash (AIA) of feed and feces was implemented as an internal indicator for the digestibility of nutrients [25].

Blood samples (about 10 mL) were collected from the jugular vein at 0 and 4 h after morning feeding on the last day of the experiment. A chemical analyzer (Mindray BS‐600, Shenzhen, China) was utilized to measure glucose, total protein, cholesterol, and blood urea nitrogen (BUN).

### 2.5. Statistical Analysis

Data were analyzed as a CRD using the general linear model (GLM) procedure of SAS software (Version 6.25) [26]. The data were examined using the following model: *Yij* = *μ* + *αi* + *εij*, where *Yij* is the observation, *μ* is the overall mean, *αi* is the treatment effect (*i* = 1–3), and *εij* is the residual error. Prior to analysis, normality and homogeneity of variance were assessed using the Shapiro–Wilk and Levene’s tests, respectively (GLM procedure). Additionally, potential outliers within treatments were screened via boxplot inspection (BOXPLOT procedure), with outliers defined as values exceeding 1.5 times the interquartile range (1.5 × IQR) away from the first (Q1) or third (Q3) quartiles; however, none were detected. Treatment means were compared using Duncan’s Multiple Range Test when significant effects were detected. Linear and quadratic effects of increasing RUSKEP supplementation levels were evaluated using orthogonal polynomial contrasts. Statistical significance was declared at *p* < 0.05. All statistical tests were two‐sided.

## 3. Results

### 3.1. Chemical Composition of Diets

The TMR can be produced from local feed resources, which contain 10.5% CP, 2.0% EE, and 32.6% NDF (Table 2). The RUSKEP contains 16.5% CP and 26.1% EE (Table 1). Moreover, the RUSKEP showed a higher C18 UFA, including oleic acid (OA, C18:1 cis‐9), LA, and ALA. The RUSKEP had higher amounts of UFA and PUFA, while the saturated fatty acids (SFAs) were lower.

### 3.2. Feed Intake and Growth Performance

The DM intake was similar between treatments (*p* > 0.05) (Table 3). Adding RUSKEP had no effect on the initial and final BW (*p* > 0.05). The ADG increased with the supplementation of RUSKEP (*p* = 0.03). Adding RUSKEP decreased FCR (*p* = 0.04). The addition of RUSKEP did not impact the feed cost per gain (FCG) (*p* > 0.05).

**TABLE 3:** Effect of rubber seed kernel pellet (RUSKEP) supplementation on feed intake and growth performance in beef cattle.

Item	RUSKEP (g per animal per day)			SEM	Contrast	
	0	200	400		*L*	*Q*
DM intake						
kg/day	5.93 (±0.84)	6.73 (±1.05)	7.03 (±1.07)	0.21	0.12	0.65
% of body weight	2.29 (±0.09)	2.38 (±0.07)	2.42 (±0.06)	0.04	0.31	0.27
Body weight (kg)						
Initial	282.30 (±17.52)	283.51 (±14.72)	291.00 (±18.94)	10.66	0.51	0.87
Final	316.73 (±15.01)	346.04 (±23.01)	343.52 (±21.63)	10.09	0.26	0.41
Growth performance						
ADG (kg/day)	0.57 (±0.36)^b^	1.04 (±0.35)^ab^	0.88 (±0.12)^b^	0.04	0.06	0.03
FCR	10.36 (±1.78)^a^	6.49 (±1.51)^b^	7.97 (±1.36)^b^	0.48	0.04	0.29
FCG (USD/kg)	1.8 (±0.30)	1.4 (±0.05)	1.5 (±0.25)	0.15	0.33	0.39

Abbreviations: ADG, average daily gain; FCG, feed cost per gain; FCR, feed conversion ratio; *L*, linear; *Q*, quadratic; SEM, standard error of the mean.

^a,b^Values in the same row with different superscripts differ according to *p* < 0.05.

### 3.3. Nutrient Intake and Digestibility

The dietary treatments had no effect on the intake of organic matter (OM), CP, NDF, and ADF (*p* > 0.05), while the EE content increased with increasing levels of RUSKEP (*p* < 0.001) (Table 4). The supplementation of RUSKEP did not influence the digestibility of DM, OM, NDF, and ADF (*p* > 0.05). However, digestibility of CP and EE was increased when cattle were fed with RUSKEP supplementation (*p* ≤ 0.03).

**TABLE 4:** Effect of rubber seed kernel pellet (RUSKEP) supplementation on nutrient intake and digestibility in beef cattle.

Item	RUSKEP (g per animal per day)			SEM	Contrast	
	0	200	400		*L*	*Q*
Nutrient intake (kg/day)						
Organic matter	5.20 (±0.74)	5.92 (±0.92)	6.19 (±0.95)	0.19	0.11	0.65
Crude protein	0.63 (±0.09)	0.72 (±0.11)	0.76 (±0.11)	0.02	0.07	0.67
Ether extract	0.12 (±0.02)^c^	0.18 (±0.02)^b^	0.22 (±0.02)^a^	0.004	< 0.001	0.67
Neutral detergent fiber	1.93 (±0.41)	2.19 (±0.52)	2.28 (±0.53)	0.07	0.13	0.65
Acid detergent fiber	1.13 (±0.16)	1.30 (±0.20)	1.35 (±0.20)	0.04	0.11	0.64
Digestibility coefficients (%)						
Dry matter	56.68 (±2.60)	57.32 (±1.57)	49.47 (±1.43)	0.91	0.07	0.32
Organic matter	62.26 (±2.55)	63.87 (±1.82)	55.79 (±1.20)	0.93	0.09	0.32
Crude protein	43.71 (±1.46)^b^	52.36 (±1.90)^a^	55.05 (±0.79)^a^	1.19	0.03	0.29
Ether extract	59.47 (1.97±)^b^	61.12 (±1.83)^ab^	66.23 (±0.90)^a^	0.76	0.02	0.39
Neutral detergent fiber	46.01 (±2.80)	49.40 (±2.98)	50.87 (±3.16)	0.96	0.13	0.70
Acid detergent fiber	30.09 (±2.78)	38.08 (±1.89)	36.63 (±4.07)	1.94	0.21	0.57

Abbreviations: *L*, linear; *Q*, quadratic; SEM, standard error of the mean.

^a–c^Values in the same row with different superscripts differ according to *p* < 0.05.

### 3.4. Blood Chemistry

The addition of RUSKEP did not impact the concentrations of BUN, total protein, and glucose (*p* > 0.05) (Table 5). The cholesterol concentration was increased with the increasing levels of RUSKEP (*p* ≤ 0.005).

**TABLE 5:** Effect of rubber seed kernel pellet (RUSKEP) supplementation on blood chemistry in beef cattle.

Item	RUSKEP (g per animal per day)			SEM	Contrast	
	0	200	400		*L*	*Q*
Blood urea nitrogen, mg/dL						
0 h postfeeding	9.75 (±4.60)	7.25 (±3.10)	11.25 (±2.22)	0.63	0.44	0.08
4 h postfeeding	10.25 (±4.15)	8.25 (±2.22)	11.25 (±2.51)	0.73	0.65	0.21
Total protein, g/dL						
0 h postfeeding	7.00 (±0.42)	6.65 (±0.81)	6.50 (±0.59)	0.14	0.30	0.83
4 h postfeeding	7.02 (±0.41)	6.85 (±0.51)	6.65 (±0.50)	0.18	0.49	0.94
Glucose, mg%						
0 h postfeeding	40.25 (±3.34)	38.75 (±3.59)	43.00 (±2.12)	1.58	0.56	0.49
4 h postfeeding	57.00 (±2.79)	56.50 (±4.73)	55.50 (±33.75)	2.10	0.81	0.96
Cholesterol, mg/dL						
0 h postfeeding	130.25 (±18.56)^c^	168.25 (±19.09)^b^	202.25 (±10.58)^a^	5.78	0.003	0.89
4 h postfeeding	132.50 (±20.55)^c^	175.75 (±25.15)^b^	210.75 (±15.31)^a^	5.73	0.005	0.78

Abbreviations: *L*, linear; *Q*, quadratic; SEM, standard error of the mean.

^a–c^Values in the same row with different superscripts differ according to *p* < 0.05.

## 4. Discussion

The chemical composition of a feed serves as a crucial nutrient source for ruminant production. The content of CP (16.5%) and EE (26.1%) in RUSKEP is similar to the results of our previous in vitro studies [19]. The content of UFA, monounsaturated fatty acids (MUFAs), and PUFA in RUSKEP was 80.31%, 24.81%, and 55.50%, respectively. This study agrees with our previous investigations, which revealed that the rumen‐protected rubber seed oil contained 81.16% UFA, 28.38% MUFA, and 52.77% PUFA [15]. The nutritional value of RUSKEP suggests its potential as a protein and lipid source for ruminants.

Adding significant levels of fat to the ruminant may have a negative impact on palatability and reduce the intake of feed. Our previous investigation of the buffaloes fed RUSKEP did not affect DM intake in swamp buffaloes [20]. Moreover, the RUSKEP supplementation had no effect on nutrient intake (OM, CP, NDF, and ADF). However, adding RUSKEP, which is rich in fats, could increase EE intake. Similarly, our previous studies reported that the inclusion of yeast‐fermented rubber seed kernel increased the intake of EE in dairy cattle [13, 14]. These results indicated that adding RUSKEP up to 400 g/animal/day did not influence the intake of nutrients in cattle.

The cattle fed with RUSKEP demonstrated an increase in EE digestibility. This finding is consistent with our previous research, which indicated that the digestibility of EE in swamp buffalo increases with the addition of RUSKEP [20]. These results may be attributed to the increased EE content of RUSKEP, which provided a higher rate of hydrolysis in the rumen when compared to the control diet. Adding RUSKEP increased the CP digestibility. These effects may result from using heat during pelleting, which reduces CP degradation and enhances CP digestibility for cattle. Dietary lipids rich in UFA are toxic to rumen microorganisms, particularly fibrolytic bacteria, thereby reducing the fiber digestibility of ruminants [27, 28]. Adding lipids, which are high in UFA, to the diets of ruminants reduced their NDF and ADF digestibility, as found in previous studies [29–31]. In the present study, RUSKEP supplementation had no influence on NDF and ADF digestibility. This effect could be due to the lower levels of RUSKEP added to the cattle, which did not influence the digestibility of fiber. This suggests that the addition of RUSKEP provides suitable levels for the nutrient digestibility of cattle.

Ruminants’ BUN concentration is employed to monitor the protein status of animal production [32]. Cattle fed with RUSKEP had no impact on the concentration of BUN. This result agrees with Ouppamong et al. [14], who reported that the increasing levels of yeast‐fermented rubber seed kernel (10%–20%) in the concentrate diet had no effect on the BUN concentration in dairy cows. Blood proteins have been utilized as common indications of nutritional and health status in animals [33, 34]. Our study found that the total protein in the blood ranged from 6.5 to 7.0 g/dL, which is within the normal range in cattle of 6.2–8.7 g/dL [3, 35, 36]. In addition, blood glucose is an important parameter for indicating energy metabolism in animals [37]. The blood total protein and glucose did not change with the RUSKEP supplementation. Similarly, our previous studies indicated that the supplementation of RUSKEP in swamp buffaloes did not alter the blood total protein and glucose concentrations [20]. The current study found that the increasing levels of RUSKEP led to a slightly increased cholesterol concentration in cattle. The addition of lipids to ruminants increased blood cholesterol levels due to enhanced absorption of long‐chain fatty acids [38], as is the case with rubber seed kernel prepared using pellets.

The RUSKEP supplementation resulted in an increase in the ADG of cattle. Adding RUSKEP at 200 and 400 g/animal/day resulted in ADG at 1.04 and 0.88 kg/day, respectively, compared to the control (0.57 kg/day). Supplementation of lipids and protein is frequently necessary to enhance the performance of cattle [39]. Adding lipids to diets enhances the energy intake and supply, which in turn promotes the accumulation of fat and the development of muscle [40]. Protein supplementation is probably the outcome of a better energy–protein balance in the rumen, which increased microbial protein synthesis and, in turn, enhanced animal growth performance [41]. RUSKEP is a beneficial source of lipids and protein. Cattle fed with RUSKEP may have increased energy and protein supply, which led to increased ADG in cattle. Another reason is that pelletizing, a mechanical process, involves the use of heat, moisture, and pressure to mix and incorporate feed‐producing pellets. Heating during pellet production creates complicated cross‐linkages between free peptides or amino acids and sugars [42]. Heat treatment of feed led to reduced degradation of protein and energy, as well as increased digestion in the small intestine [43, 44]. The addition of RUSKEP, which shifts protein and carbohydrate degradation from the rumen to the small intestine, could be responsible for these results, thereby improving the ADG in cattle. Adding RUSKEP at 200 g per animal per day shows a greater ADG than the 400 g per animal per day; however, there was no difference among treatments. The dietary UFA is toxic to the microflora of the rumen [45]. Upon UFA entry into the rumen, bacteria convert C18 UFA to C18:0; this process is commonly known as biohydrogenation [19]. Nonetheless, the rumen’s conversion of C18 UFAs to 18:0 is partial, leading to an elevated concentration of LA and ALA. Our previous studies found that the supplementation of RUSKEP up to 10% DM of substrate increased the OA, LA, and ALA in the rumen, indicating the low rumen biohydrogenation [19]. Ma et al. [46] reported that lambs fed a diet high in rumen biohydrogenation had a higher ADG compared to those fed a diet low in rumen biohydrogenation. This study shows the highest RUSKEP (400 g/animal/day), as low rumen biohydrogenation may affect the lower ADG in cattle when compared with the added 200 g/animal/day.

The FCR is an important indicator for evaluating the efficiency of feed utilization in animal production [47]. The FCR was reduced by RUSKEP supplementation. The increased amount of RUSKEP, which supply more protein and energy to the cattle, may be influencing the reduced FCR in comparison to the control group. The RUSKEP was added at 200 g/animal/day, which was the lowest FCR. This could lead to enhanced feed efficiency and increased profitability for cattle farms [48]. This suggests that adding RUSKEP, specifically 200 g/animal/day, optimizes feed efficiency and growth performance. This study utilized a small number of samples for beef cattle (*n* = 4 per treatment), possibly indicating a significant limitation. The influence of this experiment on the feed utilization and growth performance of cattle could be more significant if the application of RUSKEP remained constant over a larger sample size.

## 5. Conclusions

Supplementation of RUSKEP at 200 g/animal/day improves ADG and feed efficiency in tropical beef cattle. RUSKEP, an agricultural byproduct, can be incorporated into tropical cattle systems. Furthermore, circular economy strategies generate enhanced environmental outcomes, thereby presenting an important opportunity to advance sustainability in animal nutrition. The impact of adding RUSKEP on the meat quality and PUFA profile of beef cattle meat requires further investigation.

## Author Contributions

Pongsatorn Gunun: conceptualization, methodology, formal analysis, investigation, conducting and sampling, and writing the original draft manuscript. Walailuck Kaewwongsa, Natthapong Morthong, and Ruttanachira Ruttanaprasert: methodology and conducting and sampling. Chatchai Kaewpila and Waroon Khota: formal analysis. Nirawan Gunun: project administration, conceptualization, methodology, formal analysis, investigation, conducting and sampling, and writing the original draft manuscript. All authors: reviewing and editing the final manuscript.

## Funding

This research project was supported by Science Research and Innovation Fund, Contract no. 2566FF02TE02‐AN2.

## Conflicts of Interest

The authors declare no conflicts of interest.

## Data Availability

The datasets obtained and analyzed during the present study are available from the corresponding author upon reasonable request.
